# Using a Digital Neuro Signature to measure longitudinal individual-level change in Alzheimer’s disease: the Altoida large cohort study

**DOI:** 10.1038/s41746-021-00470-z

**Published:** 2021-06-24

**Authors:** Irene B. Meier, Max Buegler, Robbert Harms, Azizi Seixas, Arzu Çöltekin, Ioannis Tarnanas

**Affiliations:** 1 Chione GmbH, Binz, Switzerland; 2 Altoida Inc., Houston, TX USA; 3 NYU Grossman School of Medicine, Department of Population Health, Department of Psychiatry, New York, NY USA; 4 University of Applied Sciences and Arts Northwestern Switzerland, Windisch, Switzerland; 5 Global Brain Health Institute, San Francisco, CA USA; 6 Trinity College Dublin, College Green, Dublin, Ireland; 7 Hellenic Initiative Against Alzheimer’s Disease, BiHeLab Ionian University, Kerkira, Greece

**Keywords:** Prognostic markers, Alzheimer's disease

## Abstract

Conventional neuropsychological assessments for Alzheimer’s disease are burdensome and inaccurate at detecting mild cognitive impairment and predicting Alzheimer’s disease risk. Altoida’s Digital Neuro Signature (DNS), a longitudinal cognitive test consisting of two active digital biomarker metrics, alleviates these limitations. By comparison to conventional neuropsychological assessments, DNS results in faster evaluations (10 min vs 45–120 min), and generates higher test-retest in intraindividual assessment, as well as higher accuracy at detecting abnormal cognition. This study comparatively evaluates the performance of Altoida’s DNS and conventional neuropsychological assessments in intraindividual assessments of cognition and function by means of two semi-naturalistic observational experiments with 525 participants in laboratory and clinical settings. The results show that DNS is consistently more sensitive than conventional neuropsychological assessments at capturing longitudinal individual-level change, both with respect to intraindividual variability and dispersion (intraindividual variability across multiple tests), across three participant groups: healthy controls, mild cognitive impairment, and Alzheimer’s disease. Dispersion differences between DNS and conventional neuropsychological assessments were more pronounced with more advanced disease stages, and DNS-intraindividual variability was able to predict conversion from mild cognitive impairment to Alzheimer’s disease. These findings are instrumental for patient monitoring and management, remote clinical trial assessment, and timely interventions, and will hopefully contribute to a better understanding of Alzheimer’s disease.

## Introduction

**Fig. 1:**
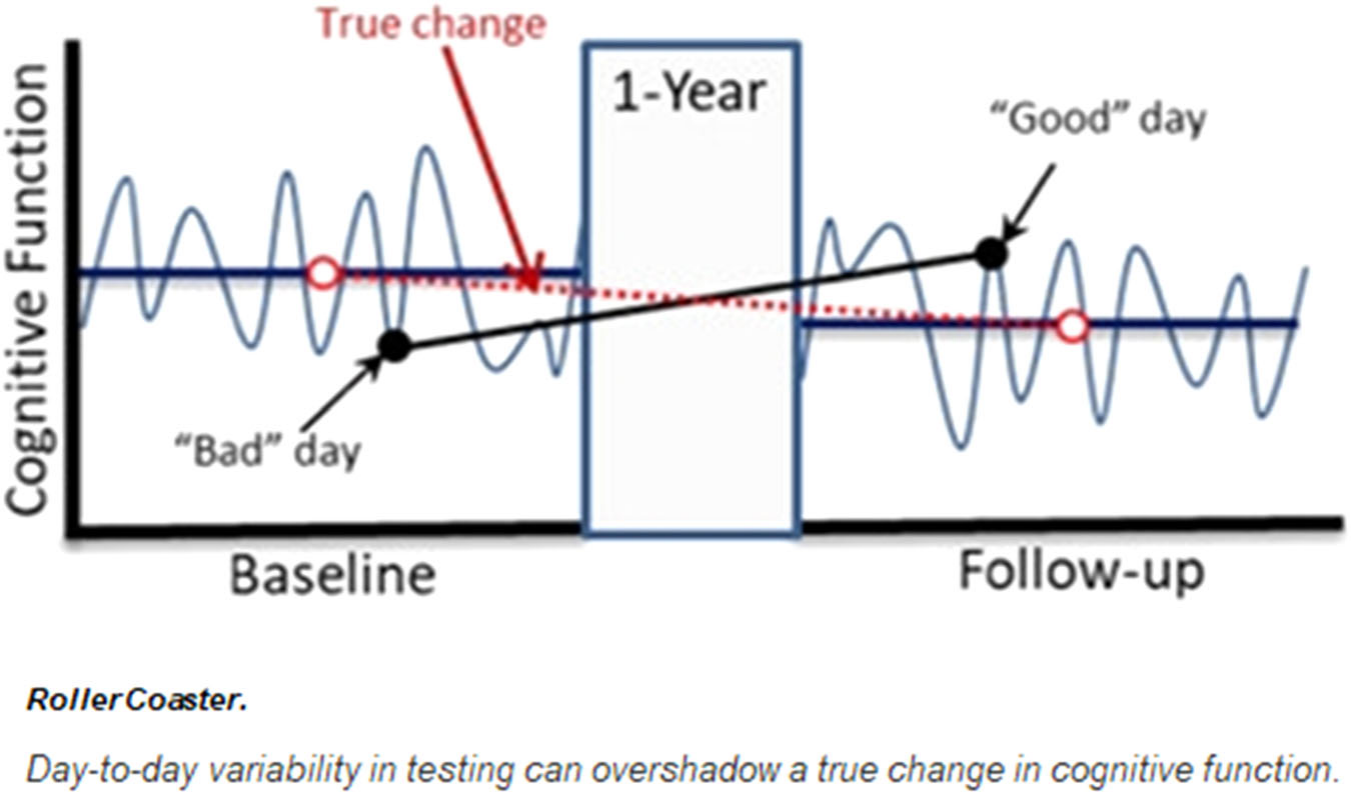
Day-to-day variability in testing can overshadow true performance due to external factors (environment) and internal factors (anxiety, motivation, etc.). Reprint courtesy of Martin Sliwinsk, permission granted^44^.

Longitudinal measures of cognitive performance are important for evaluating preclinical markers and prodromal periods of cognitive impairment and dementia, as well as for monitoring disease progression. Current techniques for assessing cognitive decline are often based on cross-sectional assessments (i.e., observations at a specific point in time). However, cross-sectional assessments are of limited value in capturing an individuals’ global cognitive function and may not accurately predict future cognitive performance and risk of cognitive decline due to high intraindividual variability (IIV) in cognitive performance^1^. Conventional cross-sectional neuropsychological assessments of cognition (NP) are vulnerable to several confounders that can affect an individual’s assessment performance, such as motivation, attention, mood, and testing environment. In turn, the unreliable nature of NPs has negative consequences for clinical care as it is used for prognosis, diagnosis, and eventually, treatment of brain-related diseases, such as the dementia family of diseases (e.g., Alzheimer’s disease (AD)). NP assessments for AD are lengthy, unreliable, and inaccurate at capturing mild cognitive impairment (MCI), and present significant variability across different contexts and times, especially after repeated measurements. Reasons for this variability are multiple, such as participants’ motivation, attention, mood, anxiety levels, sleep quality the night before the assessment, and testing environment^2^. Such variability can lead to inaccurate diagnosis and inappropriate treatment, for example, by giving the false impression that a patient’s cognition has improved at a follow-up visit (Fig. 1).

Limitations in NP highlight a significant clinical and research gap in cognitive assessment across the full spectrum of individuals from healthy cognitive function to dementia. Filling this gap is relevant to many steps of the cognitive health care pipeline from early identification of disease, to patient counseling, risk stratification, and disease management. A strong candidate in filling this gap is examining *longitudinal assessments* of cognition and cognitive decline, as observations over time may enable evaluation of preclinical markers and more accurate monitoring of disease progression. Such longitudinal assessments include two types of measurements: the delineation of the relative temporal trajectories of specific cognitive measures, and the fluctuation of cognitive performance over time within and across cognitive domains. Although longitudinal assessments may offer improved measurements of cognitive decline due to any reason (e.g., trauma and tumors), this study focuses on age-related cognitive decline. This ranges from healthy aging to MCI to the dementia family of diseases, including AD. The utility of cognitive markers (i.e., measurable variables that may capture cognitive health) in evaluating AD progression depends on the stage of cognitive decline and may vary across different disease stages^3^. However, IIV, and its dispersion (IIV across several cognitive tests), are sensitive markers for detecting change even at prodromal stages of the disease^4^.

Predicting whether the cognitive abilities of individuals with MCI will remain stable or decline (and at what rate), is challenging due to patient heterogeneity—especially when cross-sectional approaches are used. Heterogeneity makes prediction more difficult because prediction relies on establishing prior patterns and extrapolating from these patterns. For instance, while amnestic MCI (aMCI) is considered a prodromal stage of AD, symptom presentation and patterns of cognitive decline progression are not uniform and depend on several factors, such as the presence of brain atrophy, amyloid deposition, presence of the ApoE4 allele, comorbid depression, and the existence of other cognitive dysfunctions^5,6^. To overcome the problems of heterogeneity, longitudinal symptom history data are necessary, which provides an enhanced resolution of AD phenotypic variation and allows the establishment of temporal patterns in behavior in observed domains. Disease progression monitoring and prediction of conversion from “normal to MCI” or “MCI to AD” both require a granular view of individual change over time.

Research on IIV focuses primarily on performance variability as expressed by reaction time^7,8^. Latency-based measures, such as reaction time variability are well-suited for IIV research, since test scores are spread across greater time ranges than with performance differences used in traditional NP, which renders latency-based measures more sensitive. Further, reaction time is easier to measure than other, more complex measures, and it is relatively straightforward to obtain a baseline for, including through a motor test. Reaction time also allows for collecting multiple performance samples given that it is less sensitive to retest effects than, for instance, accuracy in a task which can be improved with practice^9^. High levels of IIV in reaction time have been shown to predict impending cognitive decline and are associated with a range of age-related neurological disturbances, neurodegenerative disease, and increased mortality risk^7^. Current IIV approaches take potential confounders into consideration too, such as motivation and attention that can affect cognition over time. IIV can also use accuracy-based measures in which participants execute tasks that have correct or incorrect solutions^10^. Scores obtained from accuracy-based measurements can be predictive in differentiating between patients with AD, Parkinson’s disease, and healthy controls (HC), and can help detect prodromal AD^11,12^. However, accuracy-based IIV observations are not persistent across age groups when mean cognitive performance is controlled for, which is why reaction time-based measures are preferable^13^.

**Fig. 2:**
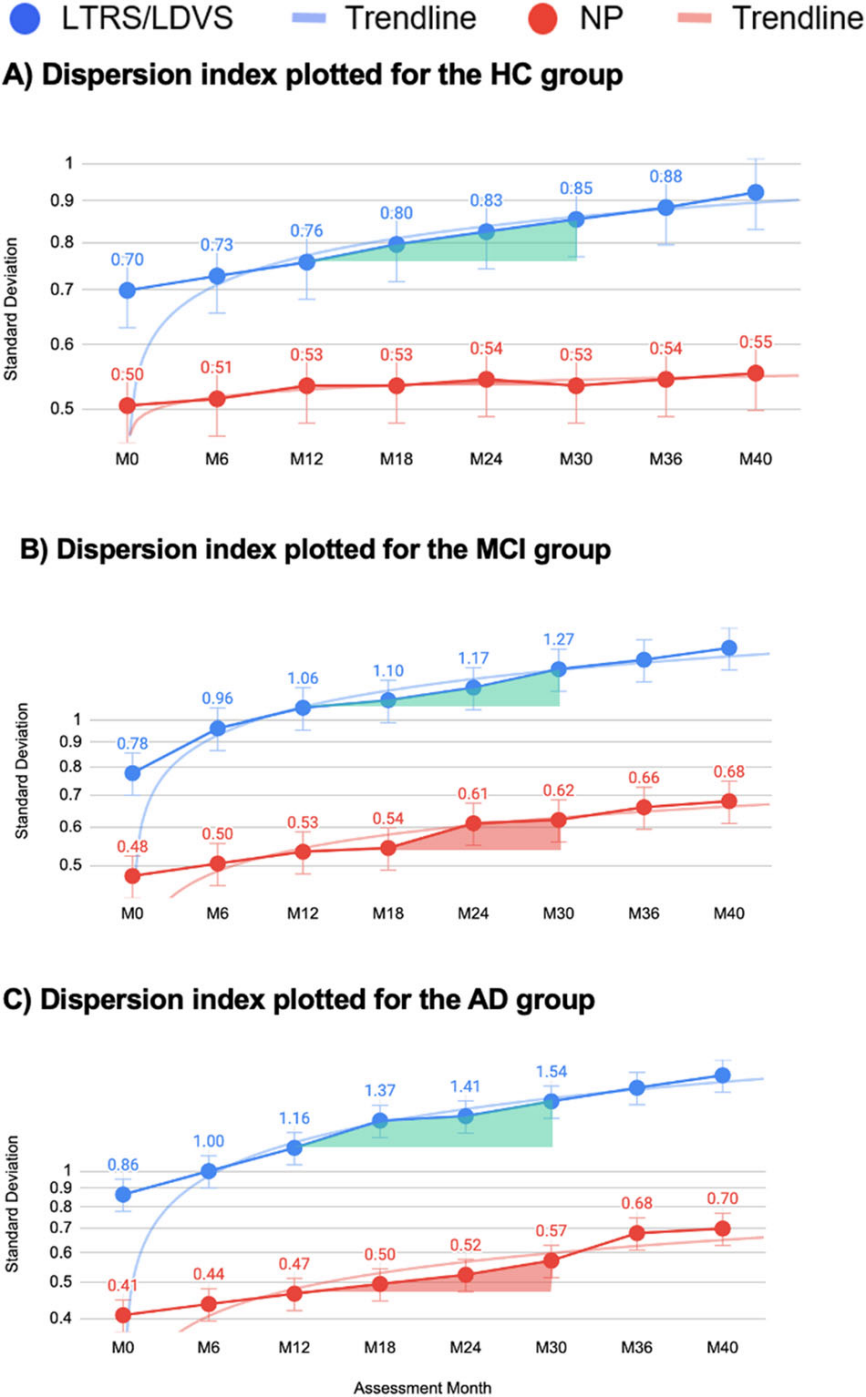
Dispersion index based on LTRS and NP plotted for for the HC (**A**), MCI (**B**), and AD (**C**) groups translated into standard deviation. The **A**–**C** graphs show a nonlinear increase in standard deviation as a function of disease trajectories. Comparing the overall mean of LTRS vs NP per group yields the following values: HC: *t* = 10.00106, *p* < 0.00001; MCI: *t* = 7.02195, *p* < 0.00001; AD: *t* = 6.65272, *p* = 0.000011, the results are statistically significant at *p* < 0.001. Details of the individual time points are shown in Table 1.

A *dispersion*-based methodology, one that analyzes multiple tasks and domains for a given individual for a complex activity of daily living, is even more promising than a single-track IIV approach in terms of understanding changes to an individual’s cognitive health over time, and in terms of establishing a precision medicine approach to dementia. Dispersion (Fig. 2) is sensitive to age-specific cognitive differences in late-life in multiple domains, especially among old-old adults (75–92 yrs) who demonstrate higher levels of dispersion than young-old adults (65–74 yrs)^14^. When extending the dispersion model to longitudinal data across multiple time points, it is possible to assess meaningful individual-level change, analogous to approaches that have been suggested using data-driven Reliable Change Index (RCI) scores^15^.

Technological advances offer solutions to the challenges of early identification of impairment in cognitive and functional abilities, and estimation of the risk of developing AD^16^. For instance, the near-continuous passive data collection from mobile device sensors allows for sensitive assessments of even subtle changes in cognitive performance. Such low-friction, passive monitoring approaches lend themselves well for longitudinal IIV (LIIV) analyses. Previous work using high frequency and passive digital phenotyping approaches show reasonable utility in differentiating symptomatic patients from HC based on features computed purely from device data. This includes the Apple-Evidation MCI study using the Apple Watch with a sleep monitor^17^, and the IBM voice analysis study^18^. Physiological and behavioral observations collected via passive or active use of digital tools (computers, smartphones, tablets, wearables, etc.), may contain indicators of disease, termed *digital biomarkers*. For cognition studies, digital biomarkers offer a novel method to capture high-dimensional data that enables examining many different variables together. It also offers an improved understanding of cognition from multiple perspectives. Digital biomarkers not only allow for active and frequent measurements of reaction time and accuracy-based scores, but also offer objective and reliable quantification of passive physiological and behavioral inputs (and lack of inputs), such as tremor, hesitation, gait, and touch pressure. While traditional tests typically capture one item per question, device monitoring can generate countless digital biomarkers with less patient burden.

One such active digital biomarker tool is the Altoida Digital Neuro Signature (DNS), previously known as the Altoida Neuro Motor Index. This tool assesses a user based on a battery of tests provided over the course of 10 min, including with respect to eye tracking, motor, and augmented reality-based tests. Altoida DNS evaluates cognition, motor skills, and function, and it predicts the risk of developing MCI due to AD. Aside from its 94% prognostic accuracy, the Altoida DNS is a highly sensitive (0.91) and specific (0.82) tool to measure dementia disease progression^19^. The Altoida DNS collects several additional variables to the conventional latency- and accuracy-based IIV, such as gait and touch pressure, which are categorized into 11 everyday function/cognition domains, which correspond to the major neurocognitive networks^20^, such as perceptual motor coordination, complex attention, and cognitive processing speed^19^. In this manuscript, we examine how dispersion measured with the Altoida DNS compares to the NP for disease monitoring, characterizing longitudinal risk trajectories, and predicting cognitive conversion events (from healthy to MCI, from MCI to AD).

## Results

### Risk trajectory-related metrics

The dispersion score across the entire sample was 11.45 (SD = 5.12) *T* score units. Figure 2 shows the magnitude of dispersion within each cognitive status subgroup (HC, MCI, and AD) based on longitudinal trajectory risk scores (LTRS) and NP, demonstrating that the digital biomarkers explain up to 2.6 times more IIV compared to conventional paper–pencil NP assessments.

Inferential analysis on the comparison of the values shown in Fig. 2 reveals that IIV differs between groups (*F*(2,522) = 34.252, *p* < 0.001, *η*^2^ = 0.25), where the AD group (*m* = 23.78, SD = 4.54) exhibits the highest variability, followed by the MCI (*m* = 12.48, SD = 2.91), and the HC (*m* = 8.09, SD = 1.64). We also observed group differences across all domains based on the battery of 13 NP vs 11 Altoida DNS domains (Table 1). The AD group exhibited greater dispersion than MCI, and MCI greater than the HC (Table 1), verifying the robustness of measurements.

**Table 1:** LTRS/LVDS vs NP trajectory differences and effect sizes (Cohen’s *d*).

Healthy LTRS/LVDS vs NP	*t* Value	*p* Value	Cohen’s *d*	95% CI		MCI LTRS/LVDS vs NP	*t* Value	*p* Value	Cohen’s *d*	95% CI		AD LTRS/LVDS vs NP	*t* Value	*p* Value	Cohen’s *d*	95% CI	
M0–M6	10.37	0.009	1.1893	0.9438	1.4349	M0–M6	4.06	0.056	0.5742	0.2913	0.857	M0–M6	7.33	0.018	1.639	1.1325	2.1456
M6–M12	12.48	0.006	1.4411	1.1871	1.6951	M6–M12	9.67	0.011	1.3675	1.0597	1.6754	M6–M12	7.92	0.016	1.771	1.2539	2.288
M12–M18	12.50	0.006	1.4434	1.1893	1.6975	M12–M18	26.92	0.001	3.8071	3.3423	4.2718	M12–M18	7.25	0.018	1.6211	1.116	2.1263
M18–M24	13.82	0.005	1.5958	1.3359	1.8556	M18–M24	11.52	0.007	1.6292	1.3093	1.949	M18–M24	36.20	0.001	8.0946	6.766	9.4232
M24–M30	28.17	0.001	3.2528	2.9079	3.5977	M24–M30	11.23	0.008	1.5882	1.2703	1.906	M24–M30	12.85	0.006	2.8733	2.2486	3.4981
M30–M36	22.77	0.002	2.6293	2.3203	2.9383	M30–M36	18.86	0.003	2.6672	2.2862	3.0482	M30–M36	11.40	0.008	2.5491	1.9591	3.1391
M36–M40	17.71	0.003	2.045	1.7657	2.3242	M36–M40	17.46	0.003	2.4692	2.1013	2.8372	M36–M40	15.41	0.004	3.4458	2.755	4.1365

### Intraindividual variability-related metrics

**Fig. 3:**
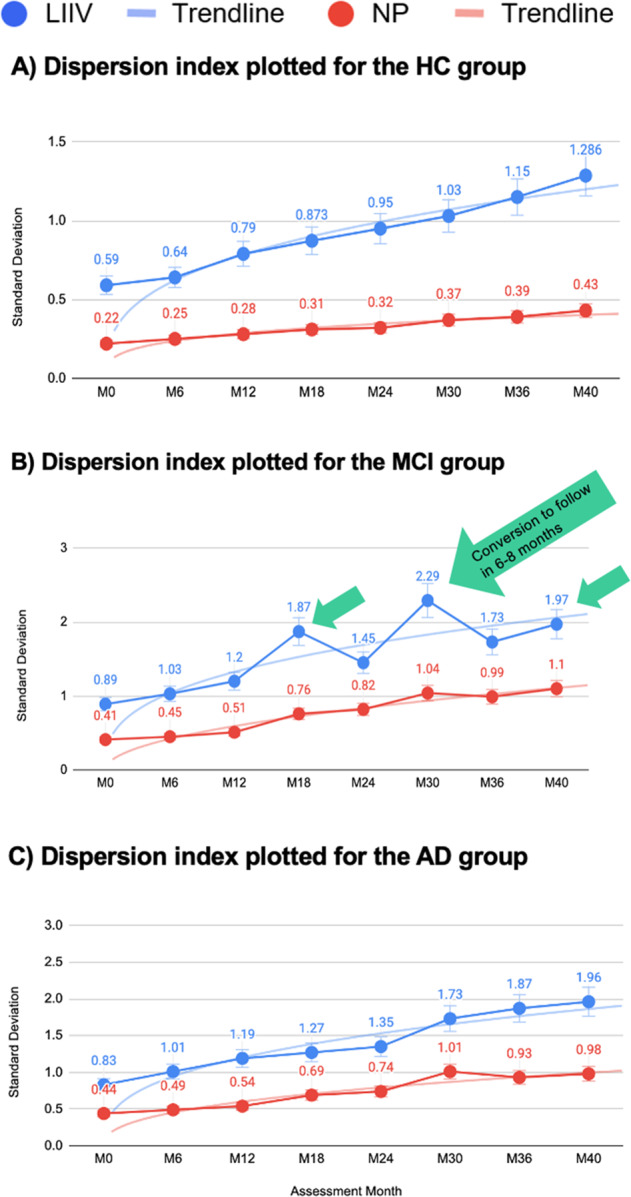
Dispersion index plotted across tasks, showing group intraindividual standard deviation (iSD) for the HC (**A**), MCI (**B**), and AD (**C**) groups. The **A**–**C** graphs show a nonlinear increase in SD as a function of disease trajectories. IIV is consistently and significantly more sensitive for the disease trajectory trends than conventional NP assessments, especially at the pre-conversion events (spikes in **B** predict a likely conversion by next assessment). LIIV longitudinal IIV.

Following the LTRS/longitudinal decline velocity scores (LDVS) analysis, we also plotted the LIIV for each group revealing a nonlinear increase in standard deviation as a function of disease trajectory (Fig. 3). In Fig. 3, IIV is consistently and significantly more sensitive at detecting disease trajectory trends than conventional NP assessments, especially with pre-conversion events (spikes in Fig. 3B predict a likely conversion from MCI to AD by next assessment). The “distance” in dispersion measures increases between the two assessment types (LIIV and NP) as the disease progresses, demonstrating that LIIV is more sensitive at detecting the markers than NP (Fig. 3).

### Longitudinal Digital Neuro Signatures

Since Altoida DNS contains both LTRS and LIIV measures, the results shown in Figs. 2 and 3 provide evidence of the strength of digital biomarkers. LIIV is a strong preclinical risk predictor that determines conversion from MCI to AD (Fig. 3B) through machine learning algorithms, whereas the LTRS shows higher sensitivity at detecting change in cognition than conventional NPs (Fig. 2 and Table 1).

Taken together, these results demonstrate that the Altoida DNS dispersion metric is consistently and significantly more sensitive at capturing disease trajectory trends than traditional NP assessments. In addition, the Altoida DNS assessment allows for the *prediction* of conversion events 6–8 months prior to the conversion event. These conversion predictors are characterized by a spike in the IIV in the assessment prior to the actual conversion, illustrated in Fig. 3B, and are not detectable with conventional NP assessments.

## Discussion

In this study, we tackled a persistent problem in cognitive aging research; the *individual-level change* in dementia with regards to cognition and function. Establishing when meaningful individual-level change has occurred is essential for evaluating dementia interventions, as well as for supporting lifelong brain health^21^. The two metrics examined here in combination (LTRS/LDVS and LIIV integrated in Altoida DNS) may offer potential tools for practitioners. LTRS/LDVS at the individual level may be useful to assess the efficiency of cognitive training, medication, or remediation, and it is a valuable alternative to the more frequently used RCI. Provided the frequency of data collection is sufficient, LTRS/LDVS makes it possible to assess individual changes in performance more sensitively than conventional paper–pencil assessments, and without the inconvenience of having to compare with change in a normative sample subject to interindividual variability issues. Also, unlike the traditional RCI, the LIIV offers a reliable tool to draw conclusions solely based on individual performance. This may be particularly valuable in the context of adaptive trials that utilize information on an ongoing basis for the purposes of maximizing trial efficiency, as well as for early detection of disease progression events, including those in the prodromal phase of dementia^22^.

In the context of AD, dispersion has been shown to be a sensitive marker to detect change in cognition and functional abilities even at prodromal stages of the disease^3,23–25^. Establishing meaningful change at the level of an individual is instrumental, as significant effects in group-level statistics do not show (and cannot even imply) what changes have occurred for any one individual^26^. Taking both of these facts into account, we analyzed dispersion differences between a full 120–140 min conventional NP assessment with 13 cognitive domains and the 10 min Altoida DNS assessment with 11 cognitive domains, and compared the dispersion in a group of HC, MCI, and AD participants over 40 months. The Altoida DNS showed consistently and significantly higher sensitivity in capturing these changes for disease trajectory trends. This was particularly true at later stages of the disease, as shown in LTRS/LDVS results (Fig. 2 and Table 1), likely due to the complex domains integrating function and cognition uniquely in the Altoida DNS. These findings render Altoida DNS a useful tool for disease progression monitoring, as well as clinical trial endpoints. Further, IIV was consistently more sensitive at identifying markers of disease trajectory trends than the conventional NP assessment (Fig. 3). IIV was also a particularly strong marker among the Altoida DNS metrics at detecting pre-conversion events, rendering the tool capable of predicting conversion from MCI to AD 6–8 months prior to the actual event. Such a prediction not only allows for lifestyle interventions to delay conversion and maintain a healthier brain for longer, but also gives patients and family time for preparation, care adjustment, and pharmacological intervention once available. In addition, this approach can allow for prospective and longitudinal assessments of biological (imaging, genetic, and biochemical) and functional markers implicated with the pathophysiology of dementia. This should help lead to a greater understanding of the development and onset of the disease. To the best of our knowledge, the DNS longitudinal patterns are the only available metrics able to predict transition events from MCI to symptomatic AD months in advance.

The Altoida DNS differs from the conventional NP in that it captures multidimensional digital biomarkers and it is not limited to latency- or accuracy-based measures. It integrates several objectively measured features into a single task. This integration increases the ecological validity of the observations, as it creates a more generalizable “real-world situation” than the traditional laboratory test settings. It is unsurprising that the abundance of data collected by Altoida DNS both by the novel combination of multiple variables addressing 11 cognitive domains, as well as sensor data yields a higher sensitivity, particularly when variability measures are considered. Digital biomarker platforms, such as Altoida DNS, produce significant volumes of high-resolution data that include cognitive and motor processing; voice-based data that are indicative of the affective state and micro-errors that divulge where, when, and how a disease manifestation is affecting everyday function. These data have the potential to be further leveraged for disease progression modeling, for more accurate conversion event prediction or modeling of drug effects, leading to at-scale, nonintrusive lifelong monitoring of brain health.

It is important to note that both dispersion and IIV exhibit a nonlinear increase with age. Current patterns of data reveal that greater dispersion across domains is associated with poorer cognitive performance, possibly reflecting reduction in cognitive control. The spikes of IIV in the MCI group are potentially explained by the demands of executive function, a domain particularly affected in MCI, due to the complexity of the Altoida DNS assessment, in addition to internal and external factors, such as anxiety and depression that particularly affect this disease stage.

Another important feature of the Altoida DNS is its efficiency. It takes 10 min to administer the Altoida DNS as opposed to a 120 min conventional NP battery, and it yields highly comparable results when administered at home as opposed to during a clinic visit. Also, heterogeneity/homogeneity features of DNS scores and LTRS/LDVS or LIIV changes in diverse cognitive abilities may also be a valuable tool for clinicians. Our findings highlight the sensitivity of digital biomarkers at detecting changes in cognition and open interesting directions for research concerning heterogeneity in cognitive change. Further analyses of interindividual differences in the patterns of change for mixed dementias or other conditions were beyond the scope of the present study, but could help provide greater understanding of individual developmental trajectories in healthy aging and the characteristics of trajectories that might be related to unhealthy aging. For example, our participants were aged 55–90, an age range that would experience some level of age-related cognitive decline even in the healthy aging group. Future studies could analyze differences based on age groups, though it is important to note that for the purposes of this study the relative measurements (comparing Altoida DNS to conventional NP) are robust, since both methods were applied to all participants in a within subject design. Similarly, for pragmatic reasons, our participants were potentially more “tech-savvy” than average for their age group, since we included owners/active users of an iPad or iPhone (and those who had Wi-Fi at home). Familiarity with technology can introduce bias in adherence^27^ and should be considered in technology-driven solutions.

Overall, the present study demonstrates that active digital biomarkers are useful tools for monitoring disease progression in cognitive aging. Such tools could be used by primary caregivers without much training in dementia testing to refer patients for further testing, or to provide necessary resources to mitigate debilitating effects of cognitive decline. This study’s findings are also relevant to clinical trials, as the prediction of AD conversion 6–8 months prior to the event may allow the detection of meaningful change that could also influence the dosage of medication and permit closer patient monitoring. Finally, observing such changes early enables the studying of underlying disease markers immediately prior to conversion, contributing to increased understanding of pathophysiological processes of AD and the possible discovery of new phenotypes of cognitive decline.

This study represents the first attempt to explore active digital biomarkers, such as those included in the Altoida DNS, for detecting meaningful change based on newly utilized metrics at the individual level. While mean scores of cognitive tests are important for disease characterization, the IIV across tests harbors large amounts of information that can easily be captured. Novel metrics using smart-device sensors show an increased sensitivity compared to conventional NP assessments. The Altoida DNS is 2.6× more sensitive than a conventional battery for dementia and takes only 10 min. This “better” and “faster” performance renders DNS an exceptional tool for patient care and can also be used to determine when an individual has undergone meaningful change in symptoms for monitoring drug interventions.

## Methods

### Study design

We conducted two experiments (Study A and Study B) to assess Altoida DNS against a set of established NPs as baseline. Study A (ClinicalTrials.gov Identifier: NCT02050464) was a semi-naturalistic observational study that included 29 participants, age 65+, with mild to moderate AD diagnosis recruited in Hirslanden Clinic, ZH, Switzerland. Study B (ClinicalTrials.gov Identifier: NCT02843529) was also a semi-naturalistic observational multicenter study, which included 496 participants (213 MCI and 283 HC), performed in ten European memory clinics and primary care centers, and two primary care community centers in the USA. Thus, a total of 525 participants enrolled in the two studies. These participants were either cognitively healthy (*n* = 283) or diagnosed with MCI (*n* = 213) or AD (*n* = 29). The studies shared similar entry (inclusion/exclusion) criteria and clinical scales, and we characterized the AD biomarkers using the same criteria for the analysis. Both studies were approved by the local institutional review board, i.e., Bioethics committee of the Ionian University in Corfu, Greece, where the studies were initiated.

In these studies, we measured cognitive performance of the participants in three groups, namely HC, MCI, and AD, using Altoida DNS, and a set of traditional pencil-and-paper NP. Thus, in this retrospective observational analysis, our *independent variable* is the testing method, Altoida DNS vs NP (elaborated under Materials), and our key-*dependent variable* is dispersion.

### Participants

In both Study A and Study B, we excluded participants with any significant neurologic disease at the recruitment stage, such as Parkinson’s disease, Huntington’s disease, normal pressure hydrocephalus, brain tumor, progressive supranuclear palsy, seizure disorder, subdural hematoma, multiple sclerosis, or history of significant head trauma followed by persistent neurologic defaults or known structural brain abnormalities. In Study B, further key inclusion criteria were (1) 55–90 years of age, (2) fluency in English, French, Spanish, Greek, German, or Italian, and (3) familiarity with digital devices, including currently possessing and actively using an iPad Pro or iPhone with an at-home Wi-Fi network for the remote assessments. Using these criteria, we first recruited a control group of 283 cognitively healthy individuals that underwent the same procedure at the Global Brain Health Institute at Trinity College, Dublin. In recruiting participants with cognitive impairments, the biomarkers (cerebrospinal fluid (CSF), brain MRI, and ApoE genotype) were used as a criterion, and cognitive deficits compatible with MCI diagnosis were found in 213 subjects: 170 from the memory clinics and primary care centers in various countries in Europe (detailed under “Procedure” section below) and 43 from the community centers in the USA. Seven participants were excluded from the data analysis due to poor data quality. The Study B cohort consisted of HC (*n* = 283), and patients with MCI who are at high risk of developing AD within 18–40 months (*n* = 213), assessed every 6 months. The MCI and AD cohorts were included independently on their biomarker status if their diagnosis was consistent with MCI and Alzheimer’s dementia diagnosis, according to core criteria of NIA-AA revised guidelines^28^. The participant cohort in Study B is further detailed in Buegler et al.^19^. The cohort in Study A (the symptomatic AD patients from the Hirslanden Clinic, Zurich, Switzerland) was added for control and comparison (*n* = 29). Participants were matched on gender and educational level, with no statistically significant difference in cognitive performance between age groups on variables education (*p* = 0.43, Cohen’s *d* = 0.4) or gender (*p* = 0.68, Cohen’s *d* = 0.3).

### Procedure

**Fig. 4:**
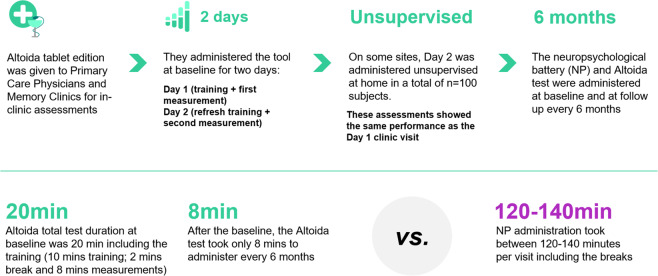
An overview of the procedure used in both studies, except for the unsupervised Altoida DNS use, which was conducted only in Study B in HCs and participants with MCI.

Upon enrollment, all participants gave written informed consent for participation and for reuse of their data. In all groups (HC, MCI, and AD), the Altoida DNS test was administered every 6–8 months over 2 days; day 1 included training and a first measurement, and day 2 included a “refresher training” followed by a second measurement. One hundred participants used Altoida DNS at home on day 2 (these measurements were verified against those obtained in the clinic before inclusion in the analysis). An overview of the procedure is represented in Fig. 4.

As shown in Fig. 4 above, the first Altoida DNS total test duration was 20 min including training (10 min training, 2 min break, and 8 min measurement). After establishing this baseline, the Altoida DNS test took an average of 8 min to administer every 6–8 months. The conventional NP assessment took between 120 and 140 min per visit, including breaks. Every 6–8 months, participants were also assessed for their clinical and NP status with the Mini-Mental State Examination (MMSE)^29^ or Montreal Cognitive Assessment (MOCA)^30^, and clinically examined if a transition from MCI to dementia (due to AD, or not associated with AD) occurred based on the diagnostic core criteria of NIA-AA^28^. Clinical outcomes for MCI/dementia/AD diagnoses were ascertained by investigators blinded to the predictor variables of this study.

Study A participants were tested for a total duration of 48 months between 2013 and 2017, and Study B participants for 40–42 months between 2017 and 2020. Participating memory clinics were in Greece, Italy, Spain, Ireland, Switzerland, and the USA. Specifically, the following institutions enabled data collection for Study B: Greek Alzheimer’s Association and Related Disorders “Ag. Giannis”, and “Ag. Eleni” memory clinics in Thessaloniki, Greece; the University of Roma La Sapienza memory clinic in Rome, Italy; IRCCS Centro San Giovanni di Dio Fatebenefratelli memory clinic in Brescia, Italy; Neuromed IRCCS memory clinic in Naples, Italy; Fundacion Clinic per a la Recerca Biomédica memory clinic in Barcelona, Spain; University of Dublin, Trinity College, St James memory clinic in Dublin, Ireland; BiHELab—Bioinformatics and Human Electrophysiology Lab and affiliated primary physicians’ network in Corfu, Greece; two offices from the Practice for Personalized Medicine of the Hirslanden Private Hospital in Zurich and Aarau, Switzerland Scripps Health in La Jolla, California, USA; and the Center for Brain Health—The University of Texas at Dallas, USA.

### Materials

The baseline NP assessments included a comprehensive set of tests: the Wechsler Memory Scale (adjusted for education)^31^, MMSE^29^ or MOCA^30^, Clinical Dementia Rating (CDR) Memory Box score^32^, and a full NP battery, including the assessments Digit Span Forward, Digit Span Backward, Trail Making Test A, Trail Making Test B^33^, RAVLT Total, RAVLT A6, RAVLT A7 (ref. ^34^), Benton VRT^35^, Digit Symbol^36^, Block Design^37^, Similarities^38^, and Word and Animal Fluency^39^. These tests, taken together, address 13 cognitive domains.

We collected the digital biomarker data for cognition and functional abilities using the Altoida DNS^19^. Altoida DNS selects the most promising indicators from previous work (such as those cited above) reducing the testing time from nearly 2 h–10 min. It also contains new measures that have not been used in this context (e.g., measuring gait, touch pressure, walk path, and tremor). This multivariate scoring increases the efficiency of digital phenotyping and enables better assessment of an individual’s performance against their own history, as well as against the “normative” data based on other people in the same cohort. The Altoida DNS captures over 320 individual features, such as reaction time, speed, attention- and memory-based assessments, as well as every single device sensor input (or lack thereof) through accelerometer, gyroscope, magnetoscope, camera, microphone, and touch screen. We piloted Altoida DNS in an independent pilot study with a sample of young, HC across all Altoida cognitive domains, and found that test-retest variability was 0.156%. Such low variability shows excellent internal validity of the Altoida DNS test and corroborates the representability and stability of its measures over time.

In addition, we collected AD biomarkers, consisting of β-amyloid and *p*-tau and total tau protein *CSF* levels, brain MRI, and ApoE genotype as specific baseline measurements for the digital biomarkers obtained through Altoida DNS test. To ensure a finer understanding of the type of cognitive impairment; classification in the diagnostic clusters of MCI and dementia due to AD (aMCI and ADD), or MCI and dementia not associated with AD (naMCI and nADD), were performed based on the β-amyloid and tau protein CSF levels biomarker.

### Statistical analyses

**Fig. 5:**
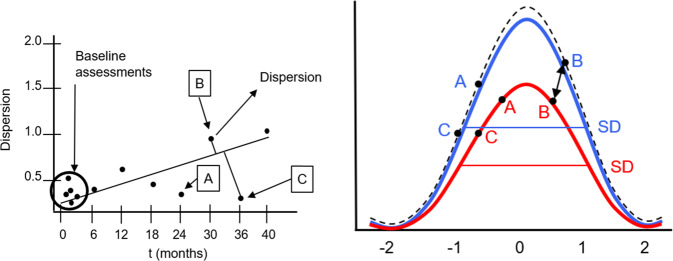
An illustration of dispersion. Left: Individual patient data over time. Right: patient performance dispersion (dots) at different time points (A, B, and C) represented in *population mean* (line) picked up using Altoida (blue, top curve) and conventional NP (red, bottom curve). SD is related to the dispersion of a given subject over time (LTRS). Black (dashed line): true dispersion.

To investigate variability in participants’ cognitive performance, a common and meaningful index that can be compared between the Altoida DNS and gold standard NP assessments is necessary. For this, we used the so-called *dispersion index*^40,41^, calculated for each individual based on their reaction times (including a control for speed-accuracy trade off) across cognitive measures within individuals and between HC, MCI, and AD groups (Fig. 5). The dispersion index is a more reliable measure of central nervous system integrity and of individual cognitive structure than mean performance^42^. Individual dispersion profiles are obtained by using a regression technique, which computes intraindividual standard deviation (iSD) scores from standardized test scores. We obtained dispersion profiles for all cognitive domains measured by the Altoida DNS and the NP test batteries used in the study to make them directly comparable. Test scores from the NP battery were initially regressed on linear and quadratic age trends to control for group differences in mean performance. Controlling for group differences based on age is necessary because greater variance tends to be associated with greater means and mean-level performance, which are expected to differ across age bands present in the study sample with participants in the age range of 55–90. The resulting residuals from these linear and quadratic regression models were standardized as *T* scores (*M* = 50, SD = 10), and iSDs were subsequently computed across these residualized test scores. The resulting dispersion estimate, indexed on a common metric, reflects the amount of variability across an individual’s NP profile relative to the group average (Fig. 5). The group average is obtained from participants’ performance levels across measurements. Higher values in the dispersion index reflect greater IIV in cognitive function.

Next, LTRS and LDVS dispersion were computed, explained in further detail below, across the 11 Altoida DNS, and the 13 conventional NP cognitive/functional domains.

For between-group mean comparisons, we used MANOVA and independent one-way ANOVA or *T* test, whereas for within-group mean comparisons, we used independent one-way ANOVA. The Benjamini–Hochberg’s correction^43^ for multiple testing was applied on all statistical analyses, using an alpha value of 0.05 (*p* < .05, two-tailed). All statistical analyses were performed using SPSS 22.0 for Mac.

### Longitudinal intraindividual variability-related metrics

**Fig. 6:**
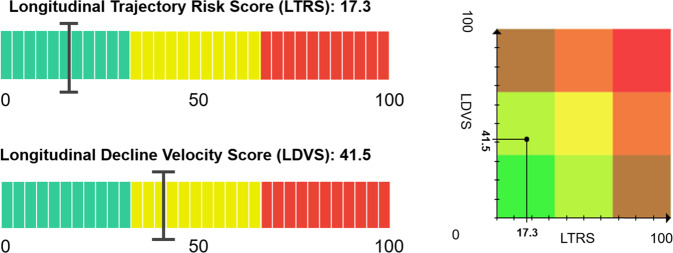
Illustrations of the LTRS (top) and LDVS (bottom) on the left (numbers 17.3 and 41.5 are random examples). The two measures can be used in a matrix to obtain a combined longitudinal risk matrix (right). Green indicates low risk, yellow medium, and red high risk. In the risk matrix (right), the overlapping areas allow for a more nuanced interpretation.

The LTRS quantifies the changes on all cognitive domains, such as the amount of cognitive decline suffered by an individual, based on multiple linear regression models (Fig. 6, top left). The LTRS does not take the period of time in which the decline occurs into account. It merely quantifies the magnitude of change that is captured by the observations. The LDVS, on the other hand, quantifies *at what speed* the change takes place, and thus can be used to assess whether decline is happening at a critical velocity in each of the cognitive domains (Fig. 6, bottom left). The LDVS is also based on multiple linear regression models. A high value in the LDVS implies an unusually fast decline and builds a weighted linear regression model for each Altoida DNS cognitive domain, using simple linear regression with the rate of decline as “weight”. The LTRS and LDVS give reliable results only when a participant performs at least four complete tests over a period of multiple weeks and can be interpreted together in a risk matrix (Fig. 6, right).

**Fig. 7:**
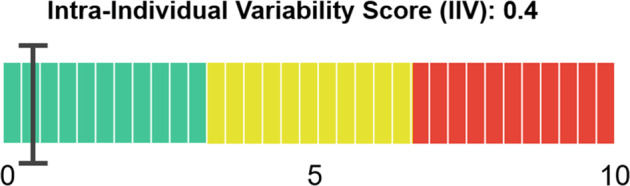
The IIV quantifies the variability of cognitive domain percentiles over time. The value corresponds to the average variability of the subject’s test in multiples of the variability of healthy subjects for each domain. The value is only reliable for at least five tests done by the same participant.

The IIV quantifies the fluctuation in cognitive performance of an individual, and has been shown to sensitively detect underlying neural pathology of cognitive and functional change at earliest stages of AD (Fig. 7). The IIV is a highly sensitive predictor of disease onset and conversion to AD.

### Reporting summary

Further information on research design is available in the Nature Research Reporting Summary linked to this article.

## Supplementary information

Reporting Summary

## Data Availability

The corresponding author (I.T.) can provide the dataset used upon reasonable request.

## Code availability

The corresponding author (I.T.) can provide the documentation on the analyses performed upon reasonable request.

## References

[1] Mungas D (2010). Heterogeneity of cognitive trajectories in diverse older persons. Psychol. Aging.

[2] Mitchell RL, Phillips LH (2007). The psychological, neurochemical and functional neuroanatomical mediators of the effects of positive and negative mood on executive functions. Neuropsychologia.

[3] Bilgel M (2014). Trajectories of Alzheimer disease-related cognitive measures in a longitudinal sample. Alzheimers Dement..

[4] Snyder PJ (2014). Assessing cognition and function in Alzheimer’s disease clinical trials: do we have the right tools?. Alzheimer’s Dement. J. Alzheimer’s Assoc..

[5] Ye BS (2013). Effects of education on the progression of early- versus late-stage mild cognitive impairment. Int. Psychogeriatr..

[6] Ye BS (2015). The heterogeneity and natural history of mild cognitive impairment of visual memory predominant type. J. Alzheimer’s Dis..

[7] Bielak A, Hultsch D, Strauss E, MacDonald S, Hunter M (2010). Intraindividual variability in reaction time predicts cognitive outcomes 5 years later. Neuropsychology.

[8] Yao C, Rich JB, Tirona K, Bernstein LJ (2017). Intraindividual variability in reaction time before and after neoadjuvant chemotherapy in women diagnosed with breast cancer. Psycho-Oncology.

[9] Salthouse T (2012). Consequences of age-related cognitive declines. Annu. Rev. Psychol..

[10] Murphy KJ, West R, Armilio ML, Craik FIM, Stuss DT (2007). Word-list-learning performance in younger and older adults: intra-individual performance variability and false memory. Aging Neuropsychol. Cogn..

[11] K„lin AM (2014). Intraindividual variability across cognitive tasks as a potential marker for prodromal Alzheimeras disease. Front. Aging Neurosci.

[12] Tractenberg RE, Pietrzak RH (2011). Intra-individual variability in alzheimeras disease and cognitive aging: definitions, context, and effect sizes. PLoS ONE.

[13] Salthouse TA, Nesselroade JR, Berish DE (2006). Short-term variability in cognitive performance and the calibration of longitudinal change. J. Gerontol. Ser. B Psychol. Sci. Soc. Sci..

[14] Hilborn JV, Strauss E, Hultsch DF, Hunter MA (2009). Intraindividual variability across cognitive domains: Investigation of dispersion levels and performance profiles in older adults. J. Clin. Exp. Neuropsychol..

[15] Mella N (2018). Individual differences in developmental change: quantifying the amplitude and heterogeneity in cognitive change across old age. J. Intell..

[16] Jeff Cummings Early Alzheimer’s disease: developing drugs for treatment guidance for industry. https://www.fda.gov/files/drugs/published/Alzheimer%E2%80%99s-Disease---Developing-Drugs-for-Treatment-Guidance-for-Industy.pdf (2018). 10.1016/j.trci.2018.11.004 PMC6804505 31650002

[17] Chen, R. et al. Developing measures of cognitive impairment in the real world from consumer-grade multimodal sensor streams. In *Proceedings of the 25th ACM SIGKDD International Conference on Knowledge Discovery & Data Mining* (2019).

[18] Low DM (2020). Natural language processing reveals vulnerable mental health support groups and heightened health anxiety on reddit during COVID-19: observational study. J. Med. Internet Res..

[19] Buegler M (2020). Digital biomarker-based individualized prognosis for people at risk of dementia. Alzheimers Dement..

[20] Mesulam MM (1990). Large-scale neurocognitive networks and distributed processing for attention, language, and memory. Ann. Neurol..

[21] Livingston G (2017). Dementia prevention, intervention, and care. Lancet.

[22] Ritchie CW (2016). Development of interventions for the secondary prevention of Alzheimeras dementia: the European Prevention of Alzheimeras Dementia (EPAD) project. Lancet Psychiatry.

[23] Hultsch, D. F., Strauss, E., Hunter, M. A., & MacDonald, S. W. S. In *The Handbook of Aging and Cognition* (eds Craik, F. I. M. & Salthouse T. A.) 491–556 (Psychology Press, 2008).

[24] Wojtowicz M, Berrigan LI, Fisk JD (2012). Intra-individual variability as a measure of information processing difficulties in multiple sclerosis. Int. J. MS Care.

[25] Kim YJ, Cho S-K, Lee JS (2019). Data-driven prognostic features of cognitive trajectories in patients with amnestic mild cognitive impairments. Alzheimers Res. Ther.

[26] Murray AL (2021). Assessing individual-level change in dementia research: a review of methodologies. Alzheimers Res. Ther..

[27] Kivipelto M (2013). The Finnish geriatric intervention study to prevent cognitive impairment and disability (FINGER): study design and progress. Alzheimer’s Dement..

[28] Jack CR (2018). Contributors. NIA-AA Research Framework: toward a biological definition of Alzheimer’s disease. Alzheimers Dement..

[29] Folstein MF, Folstein SE, McHugh PR (1975). “Mini-mental state”. A practical method for grading the cognitive state of patients for the clinician. J. Psychiatr. Res..

[30] Nasreddine ZS (2005). The Montreal cognitive assessment, MoCA: A brief screening tool for mild cognitive impairment. J. Am. Geriatrics Soc..

[31] Chlebowski, C. in *Encyclopedia of Clinical Neuropsychology* (eds Kreutzer, J. S., DeLuca, J. & Caplan, B) (Springer, 2011).

[32] Morris JC (1993). The Clinical Dementia Rating (CDR): current version and scoring rules. Neurology.

[33] Butler M, Retzlaff P, Vanderploeg R (1991). Neuropsychological test usage. Prof. Psychol. Res. Pract..

[34] Bean J. in *Encyclopedia of Clinical Neuropsychology* (eds Kreutzer, J. S., DeLuca, J. & Caplan, B.) (Springer, 2011).

[35] Benton A (1962). The visual retention test as a constructional praxis task. Stereotact. Funct. Neurosurg..

[36] Kaufman AS (1983). Test Review: Wechsler, D. Manual for the Wechsler adult intelligence scale, revised. New York: Psychological Corporation, 1981. J. Psychoeduc. Assess..

[37] Hutt ML (1932). The Kohs block-design tests. A revision for clinical practice. J. Appl. Psychol..

[38] Drozdick, L. W., Raiford, S. E., Wahlstrom, D., & Weiss, L. G. in *Contemporary Intellectual Assessment: Theories, Tests, and Issues* 4th edn, 486–511 (The Guilford Press, 2018).

[39] Benton A (1968). Differential behavioral effects in frontal lobe disease. Neuropsychologia.

[40] Christensen H (1999). Dispersion in cognitive ability as a function of age: a longitudinal study of an elderly community sample. Aging Neuropsychol. Cogn..

[41] Cole, M. S., Bedeian, A. G., & Hirschfeld, R. R. Dispersion-composition models in multilevel research: a data-analytic framework (2010).

[42] Halliday DWR (2018). Intraindividual variability across neuropsychological tests: dispersion and disengaged lifestyle increase risk for Alzheimeras disease.. J. Intell.

[43] Hochberg Y, Benjamini Y (1990). More powerful procedures for multiple significance testing. Stat. Med..

[44] ALZFORUM. Cognitive testing is getting faster and better https://www.alzforum.org/news/conference-coverage/cognitive-testing-getting-faster-and-better (2017).

