# Supplementary material for: Using a Digital Neuro Signature to measure longitudinal individual-level change in Alzheimer’s disease: the Altoida large cohort study
Source: NPJ Digit Med. 2021 Jun 24;4:101. doi: 10.1038/s41746-021-00470-z (PMC8225898; doi:10.1038/s41746-021-00470-z)
Supplement: Supplementary file 1 — Reporting Summary [file 41746_2021_470_MOESM1_ESM.pdf]

nature research

Corresponding author(s): Ioannis Tarnanas
Last updated by author(s): 2020/12/28

Reporting Summary

Nature Research wishes to improve the reproducibility of the work that we publish. This form provides structure for consistency and transparency in reporting. For further information on Nature Research policies, see our Editorial Policies and the Editorial Policy Checklist.

Statistics

For all statistical analyses, confirm that the following items are present in the figure legend, table legend, main text, or Methods section.

n/a	Confirmed	
☐	☒	The exact sample size (n) for each experimental group/condition, given as a discrete number and unit of measurement
☐	☒	A statement on whether measurements were taken from distinct samples or whether the same sample was measured repeatedly
☐	☒	The statistical test(s) used AND whether they are one- or two-sided<br>*Only common tests should be described solely by name; describe more complex techniques in the Methods section.*
☐	☒	A description of all covariates tested
☒	☐	A description of any assumptions or corrections, such as tests of normality and adjustment for multiple comparisons
☐	☒	A full description of the statistical parameters including central tendency (e.g. means) or other basic estimates (e.g. regression coefficient) AND variation (e.g. standard deviation) or associated estimates of uncertainty (e.g. confidence intervals)
☐	☒	For null hypothesis testing, the test statistic (e.g. F, t, r) with confidence intervals, effect sizes, degrees of freedom and P value noted<br>*Give P values as exact values whenever suitable.*
☒	☐	For Bayesian analysis, information on the choice of priors and Markov chain Monte Carlo settings
☒	☐	For hierarchical and complex designs, identification of the appropriate level for tests and full reporting of outcomes
☐	☒	Estimates of effect sizes (e.g. Cohen's d, Pearson's r), indicating how they were calculated

Our web collection on statistics for biologists contains articles on many of the points above.

Software and code

Policy information about availability of computer code

Data collection	Altoida NMI
Data analysis	SPSS 22.0 for Mac

For manuscripts utilizing custom algorithms or software that are central to the research but not yet described in published literature, software must be made available to editors and reviewers. We strongly encourage code deposition in a community repository (e.g. GitHub). See the Nature Research guidelines for submitting code & software for further information.

Data

Policy information about availability of data

All manuscripts must include a data availability statement. This statement should provide the following information, where applicable:

- Accession codes, unique identifiers, or web links for publicly available datasets
- A list of figures that have associated raw data
- A description of any restrictions on data availability

The data that support the findings of this study are available from Altoida Inc. but restrictions apply to the availability of these data, which were used under license for the current study, and so are not publicly available. Data are however available from the authors upon reasonable request and with permission of Altoida Inc.

Field-specific reporting

Please select the one below that is the best fit for your research. If you are not sure, read the appropriate sections before making your selection.

☐ Life sciences ☒ Behavioural & social sciences ☐ Ecological, evolutionary & environmental sciences

For a reference copy of the document with all sections, see nature.com/documents/nr-reporting-summary-flat.pdf

Behavioural & social sciences study design

All studies must disclose on these points even when the disclosure is negative.

Study description	Quantitative prospective longitudinal study
Research sample	(1) 55-90 years of age, (2) fluency in English, French, Spanish, Greek, German or Italian, and (3) familiarity with digital devices, including currently possessing and actively using an iPad Pro or iPhone with an at-home Wi-Fi network for the remote assessments.
Sampling strategy	The MCI and AD cohorts were included independently on their biomarker status if their diagnosis was consistent with MCI and Alzheimer's dementia diagnosis according to core criteria of NIA-AA revised guidelines (Jack et al., 2011). The participant cohort in Study B (Table 2) is further detailed in Buegler & colleagues 2020. The cohort in Study A of symptomatic AD patients from the Hirslanden Clinic, Zurich, Switzerland was added for comparison (n=29).
Data collection	Using these criteria, we first recruited a control group of 283 cognitively healthy individuals that underwent the same procedure at the Global Brain Health Institute (GBHI) at Trinity College, Dublin. In recruiting participants with cognitive impairments, the biomarkers (CSF, brain MRI and ApoE genotype) were used as a criterion, and cognitive deficits compatible with MCI diagnosis were found in 213 subjects: 170 from the memory clinics and primary care centers in Europe and 43 from the community centers in the USA. Seven participants were excluded from the data analysis due to poor data quality. The Study B cohort consisted of HC (n=283) and patients with MCI who are at high risk of developing AD within 18-40 months (n=213), assessed every 6 months.
Timing	October 17, 2016 recruitment start, February 21, 2020 study completion.
Data exclusions	Seven participants were excluded from the data analysis due to poor data quality.
Non-participation	N/A
Randomization	The MCI and AD cohorts were included independently on their biomarker status if their diagnosis was consistent with MCI and Alzheimer's dementia diagnosis according to core criteria of NIA-AA revised guidelines (Jack et al., 2011).

Reporting for specific materials, systems and methods

We require information from authors about some types of materials, experimental systems and methods used in many studies. Here, indicate whether each material, system or method listed is relevant to your study. If you are not sure if a list item applies to your research, read the appropriate section before selecting a response.

Materials & experimental systems

n/a	Involved in the study	
☒	☐	Antibodies
☒	☐	Eukaryotic cell lines
☒	☐	Palaeontology and archaeology
☒	☐	Animals and other organisms
☐	☒	Human research participants
☐	☒	Clinical data
☒	☐	Dual use research of concern

Methods

n/a	Involved in the study	
☒	☐	ChIP-seq
☒	☐	Flow cytometry
☒	☐	MRI-based neuroimaging

Human research participants

Policy information about studies involving human research participants

Population characteristics	See above
Recruitment	Semi-naturalistic observational multicenter study performed in ten European memory clinics and primary care centers and two primary care community centers in the USA
Ethics oversight	Both studies were approved by the local institutional review board (IRB), Bioethics committee of the Ionian University in Corfu, Greece where the study was initiated

Note that full information on the approval of the study protocol must also be provided in the manuscript.

Clinical data

Policy information about clinical studies

All manuscripts should comply with the ICMJE guidelines for publication of clinical research and a completed CONSORT checklist must be included with all submissions.

Clinical trial registration	NCT02050464; NCT02843529
Study protocol	Clinicaltrials.gov
Data collection	Recruitment from the memory clinics and primary care centers in Europe and another sample from the community centers in the USA. Locale was English, French, Spanish, Greek, German or Italian. October 17, 2016 recruitment start and February 21, 2020 study completion.
Outcomes	Primary outcome was Sensitivity, specificity and accuracy of models for diagnosis of memory disorders and also change in Diagnostic Area Under the Receiver Operating Characteristic Curve (ROC-AUC).
